# PCC0208027, a novel tyrosine kinase inhibitor, inhibits tumor growth of NSCLC by targeting EGFR and HER2 aberrations

**DOI:** 10.1038/s41598-019-42245-3

**Published:** 2019-04-05

**Authors:** Qiuju Dong, Pengfei Yu, Liang Ye, Jianzhao Zhang, Hongbo Wang, Fangxia Zou, Jingwei Tian, Hiroshi Kurihara

**Affiliations:** 1 Department of Pharmacology, Life Science and Biopharmaceutics School, Shenyang Pharmaceutical University, Shenyang, P.R. China; 2 School of Pharmacy, Key Laboratory of Molecular Pharmacology and Drug Evaluation (Yantai University), Ministry of Education, Collaborative Innovation Center of Advanced Drug Delivery System and Biotech Drugs in Universities of Shandong, Yantai University, Yantai, P.R. China; 3 School of Pharmacy, Binzhou Medical University, Yantai, P.R. China

## Abstract

PCC-0208027 is a novel tyrosine kinase inhibitor that has a strong inhibitory effect on epidermal growth factor receptor (EGFR)- or HER2-driven cancers. The aim is to assess the anti-tumor activity of PCC0208027 and related mechanisms in non-small cell lung cancer (NSCLC). We examined the activity of PCC0208027 on various mutated EGFRs, HER2, and HER4. MTT assays, flow cytometry, and Western blotting were used to examine the effects of PCC0208027 on NSCLC cells with different genetic characteristics and relevant molecular mechanisms. Nude mouse xenograft models with HCC827, NCI-H1975, and Calu-3 cells were used to evaluate the *in vivo* anti-tumor activity of PCC0208027. Results showed that PCC0208027 effectively inhibited the enzyme activity of EGFR family members, including drug-sensitive EGFR mutations, acquired drug-resistant EGFR T790M and EGFR C797S mutations, and wild-type (WT) HER2. PCC0208027 blocked EGFR phosphorylation, thereby downregulating downstream PI3K/AKT and MAPK/ERK signaling pathways and inducing G0/G1 arrest in NSCLC cells. PCC0208027 inhibited tumor growth in mouse xenograft models of HCC827, NCI-H1975, and Calu-3 cells. In summary, our findings suggest that PCC0208027 has the potential to become an oral antineoplastic drug for NSCLC treatment and is worthy of further development.

## Introduction

Lung cancer is one of the most common cancers and is currently the leading cause of cancer-related deaths. Globally, approximately 1.6 million people die of lung cancer each year^1^. NSCLC is the most common lung cancer subtype, accounting for 80–85% of lung cancers and more than 50% of patients have stage IV disease at the time of diagnosis^1–3^. EGFR is the most common genetic driver in NSCLC development. Around 10–15% of Caucasian and 40% of Asian patients have mutations in *EGFR*^4^. Many pathogenic mutations, deletions, insertions, and duplications in *EGFR* exons 18–21 have been reported^5^. Small molecule EGFR tyrosine kinase inhibitors (TKIs) have become the mainstay targeted therapy for NSCLC patients with EGFR mutations^3,5,6^.

Erlotinib, gefitinib, and afatinib are first-line treatments for NSCLC patients with EGFR exon 19 deletion or exon 21 L858R mutations. In clinical practice, these treatments are superior to platinum-based chemotherapy, as in NSCLC patients with *EGFR* mutations, the response rate (RR) is 80% and progression-free survival (PFS) can be extended by 10–14 months^3,7–10^. However, treatment-related adverse events (AEs) such as diarrhea and rashes are often reported^11^. Importantly, patients who initially respond to these drugs will ultimately develop drug resistance after 1–2 years of PFS, leading to disease progression^12,13^. The most common acquired drug resistance mechanism is the secondary acquisition of a single missense mutation in exon 20 of the *EGFR* gene, i.e., T790M mutation, which accounts for 49–60% of the total number of patients with drug resistance^13,14^. Osimertinib, a next-generation EGFR TKI, is approved in the US for the treatment of patients with *EGFR* T790M mutation-positive inoperable or recurrent NSCLC that is resistant to EGFR TKI therapy, and for the first-line treatment of patients with inoperable or recurrent *EGFR* mutation-positive NSCLC. Unfortunately, even with initial positive responses, patients who undergo osimertinib treatment ultimately develop drug resistance. The most common mechanism for this drug resistance is the C797S mutation in exon 20 of the *EGFR* gene^15^. Currently, there are no effective therapies for targeting the EGFR C797S drug-resistant mutation. Therefore, discovering effective inhibitors for EGFR C797S drug-resistant mutations is of significant clinical value.

HER2 (also known as ErbB2) is a member of the ErbB tyrosine kinase family. Although HER2 does not have an endogenous ligand, it has been confirmed that HER2 is the preferential binding partner for other ErbB receptors, particularly EGFR. The HER2/EGFR heterodimer formed between HER2 and EGFR has greater signal transduction potential than EGFR homodimers^16^. In NSCLC, *HER2* amplification and insertion mutations in exon 20 of the *HER2* gene are regarded as oncogenic driver mutations. In addition, *HER2* amplification is also one of the mechanisms by which patients develop secondary drug resistance to EGFR TKIs^17^. Therefore, designing inhibitors that simultaneously target EGFR and HER2 receptors may have a significant impact on clinical efficacy, and may delay the occurrence of EGFR-TKI drug resistance. Kanthala *et al*. designed peptide inhibitors that inhibit the formation of HER2:HER3 and EGFR:HER2 heterodimers by disrupting protein-protein interactions and demonstrated inhibition of tumor proliferation in NSCLC cells and synergistic effects with erlotinib^18^.

**Figure 1:**
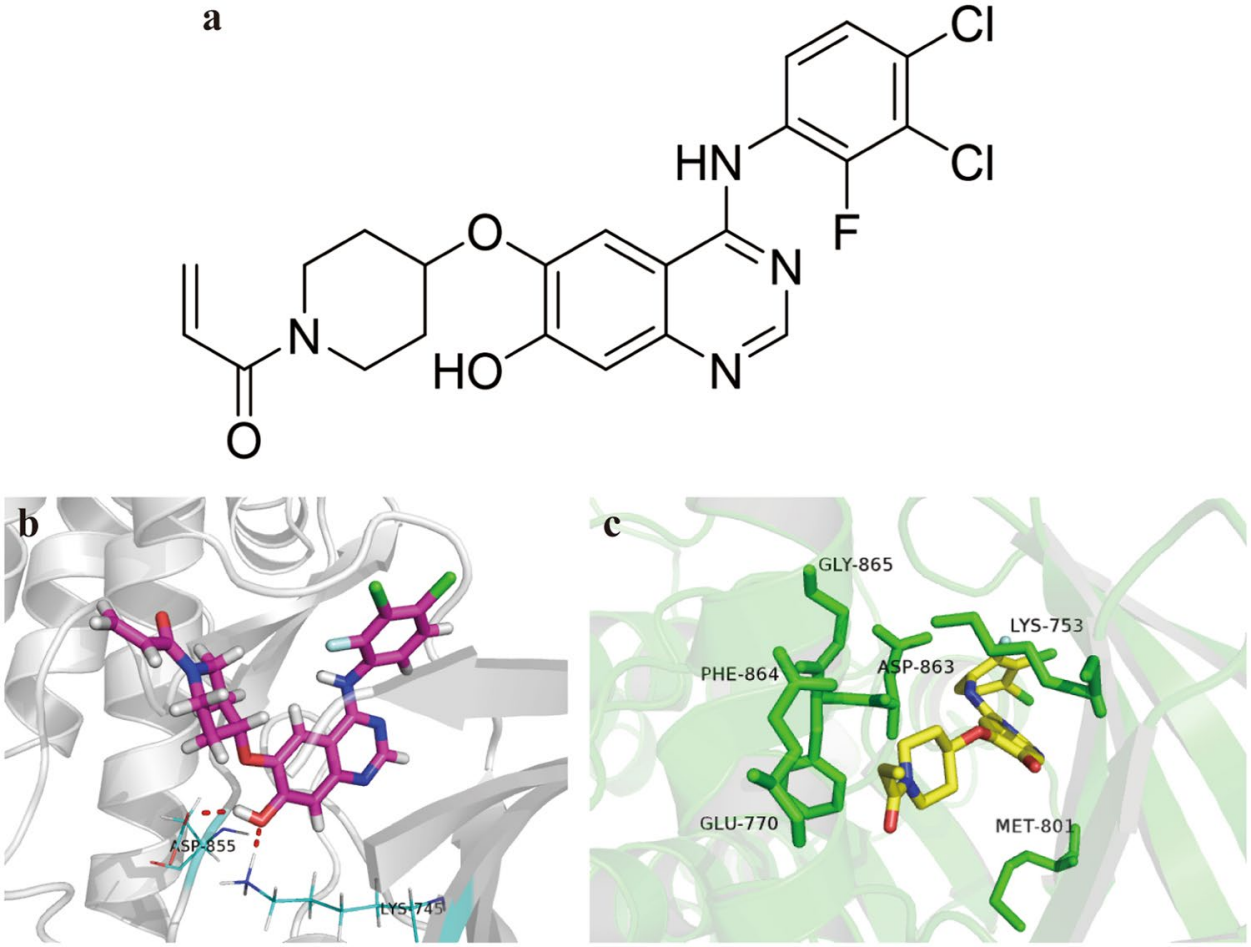
Chemical structure and binding modes of PCC0208027. (**a**) Chemical structure of PCC0208027. (**b**) Binding mode of PCC0208027 to EGFR T790M. PCC0208027 is displayed in pink, oxygen atoms are in red and nitrogen atoms in blue. Hydrogen bonds between homodimer mutant EGFR and PCC0208027 are represented as a red dash line. (**c**) Binding mode of PCC0208027 to WT-HER2. PCC0208027 is displayed in yellow, oxygen atoms are in red and nitrogen atoms in blue.

We utilized our small molecule synthesis and screening platform to synthesize a series of new compounds. We used cell-free, cell-based, and animal-based studies to comprehensively screen these compounds, and identified compound PCC0208027 (Fig. 1(a)). PCC0208027 was found to exert a potent inhibitory effect on EGFR exon 19 deletion, EGFR L858R, T790M, and C797S mutations, HER2, and HER4. Therefore, we systematically evaluated the anti-tumor activity of PCC0208027 in NSCLC tumors with drug-sensitive or -resistant *EGFR* mutations, and *HER2* amplification, and elucidated its potential anti-tumor mechanisms. This will provide more potential EGFR TKI options for NSCLC treatment.

## Results

### PCC0208027 is a potent inhibitor of EGFR family kinases

Table 1 shows the inhibitory effect of PCC0208027 on purified EGFR family tyrosine kinases. PCC0208027 significantly inhibited the drug-sensitive EGFR L858R (IC_50_ = 0.0047 nM) and EGFR d746–750 (IC_50_ = 0.0030 nM), and the drug-resistant EGFR T790M (IC_50_ = 0.61 nM), EGFR L858R/T790M (IC_50_ = 0.94 nM), EGFR d746–750/T790M (IC_50_ = 0.82 nM), and EGFR C797S (IC_50_ = 2.4 nM). PCC0208027 had a more potent inhibitory effect on the drug-sensitive EGFR mutation than erlotinib and osimertinib. PCC0208027 also demonstrated a comparable inhibitory effect on EGFR T790M mutation as osimertinib, and a strong inhibitory effect on the EGFR C797S mutation, which was resistant to osimertinib. In addition, PCC0208027 demonstrated a potent inhibitory effect on WT-EGFR (IC_50_ = 0.077 nM), WT-HER2 (IC_50_ = 0.025 nM) and WT-HER4 (IC_50_ = 0.11 nM).

**Table 1:** Inhibition of EGFR family kinases by PCC0208027.

Enzyme (IC_50_, nM)	PCC0208027	Erlotinib	Osimertinib	Poziotinib
EGFR WT	0.077	0.25	1.2	0.03
EGFR L858R	0.0047	0.36	2.1	0.057
EGFR d746–750	0.0030	0.41	1.2	0.066
EGFR T790M	0.61	443	0.30	0.65
EGFR L858R/T790M	0.94	413	0.30	1.11
EGFR d746–750/T790M	0.82	360	0.24	0.97
EGFR C797S	2.4	19	>10000	4.9
EGFR C797S/T790M/L858R	150	740	620	230
HER2	0.025	22	1.69	0.070
HER4	0.11	265	3.06	0.18

IC_50_: 50% inhibition concentration. The IC_50_ was calculated from data collected at concentrations ranging from 0.05 nM to 10 μM (n = 2).

### Binding of PCC0208027 to EGFR T790M and WT-HER2

To understand the mechanistic basis of the inhibitory effect on EGFR T790M, we analyzed the best putative binding mode of PCC0208027 by eye checking and critical residue modeling. When PCC0208027 interacted with an EGFR T790M homodimer, hydrogen bond interactions were mainly observed between the hydroxyl group of the quinazolin-7-ol core and the amine group of Met793 (Fig. 1(b)). The length of this hydrogen bond was 2.0 Å, indicating that the hydrogen bond was essential and strong for the inhibitory effect of PCC0208027 on EGFR T790M^19^.

For the docking structures of a hydroxyquinazoline analog PCC0208027 with WT-HER2, we confirmed that PCC0208027 was mapped well in the ATP pocket of WT-HER2 (Fig. 1(c)). The ethylene carbonyl group in PCC0208027 was flipped relative to its orientation in HER2/TAK-285 structures^20^. In the co-structure of PCC0208027 with HER2, the amino group in the quinazoline ring interacted with the keychain of Asp863 in the DFG motif, which is important for inhibitor potency. Moreover, the quinazoline ring can form hydrophilic interactions with the side chain of amino acids near the DFG motif. Therefore, PCC0208027 might be an effective ATP-competitive WT-HER2 inhibitor.

Additionally, we could not predict the binding mode of PCC0208027 with EGFR C797S or HER2 exon 20 mutations, as no applicable co-crystal complexes have been reported.

### PCC0208027 showed potent growth inhibitory activity in various NSCLC cell lines

Based on the effect of PCC0208027 on EGFR family members, we examined the growth inhibitory effect of PCC0208027 on different NSCLC cell lines, including EGFR-TKI-sensitive HCC827 cells, NCI-H1975 cells containing the EGFR T790M drug-resistant mutation, HER2-overexpressing Calu-3 cells, NCI-H1781 cells containing HER2 mutations and A549 cells with KRAS mutation^21^. The results (Table 2) showed that PCC0208027 can effectively inhibit the growth of various NSCLC cell lines. In the HCC827 cells that have EGFR drug-sensitive mutations, the activity of PCC0208027 (IC_50_ = 4.22 nM) was similar to erlotinib (IC_50_ = 3.71 nM), osimertinib (IC_50_ = 1.09 nM) and poziotinib (IC_50_ = 1.72 nM). In NCI-H1975 cells that contain EGFR T790M mutations, the inhibitory activity of PCC0208027 (IC_50_ = 11.11 nM), osimertinib (IC_50_ = 7.25 nM) and poziotinib (IC_50_ = 8.78 nM) were similar. However, in HER2-overexpressing or mutant NSCLC cells, the inhibitory activity of PCC0208027 (IC_50_ = 6.07 nM for Calu-3 and IC_50_ = 5.97 nM for NCI-H1781) was significantly better than erlotinib and osimertinib, and similar to poziotinib. PCC0208027 and other EGFR TKIs showed no anti-tumor activity against A549 cells with KRAS mutation.

**Table 2:** Effect of PCC0208027 on NSCLC cell viability.

Cell lines	Characterization	IC_50_ (nM)			
		PCC0208027	Erlotinib	Osimertinib	Poziotinib
HCC827	EGFR (Del E746_A750))	4.22 ± 0.51	3.71 ± 1.89	1.09 ± 0.22	1.72 ± 0.32
NCI-H1975	EGFR (L858R/T790M)	11.11 ± 4.39	>1,000	7.25 ± 2.30	8.78 ± 3.03
Calu-3	HER-2 (WT, amplified)	6.07 ± 0.29	>1,000	469.96 ± 7.30	2.47 ± 0.52
NCI-H1781	HER-2 (G776V, Cins)	5.97 ± 1.37	>1,000	842.3 ± 33.95	6.17 ± 0.42
A549	*K-ras* mutation (G12S)	>1,000	>1,000	>1,000	>1,000

Values are presented as mean ± s.d. (n = 3).

### PCC0208027 induced G0/G1 phase arrest in HCC827 and NCI-H1975 cells

**Figure 2:**
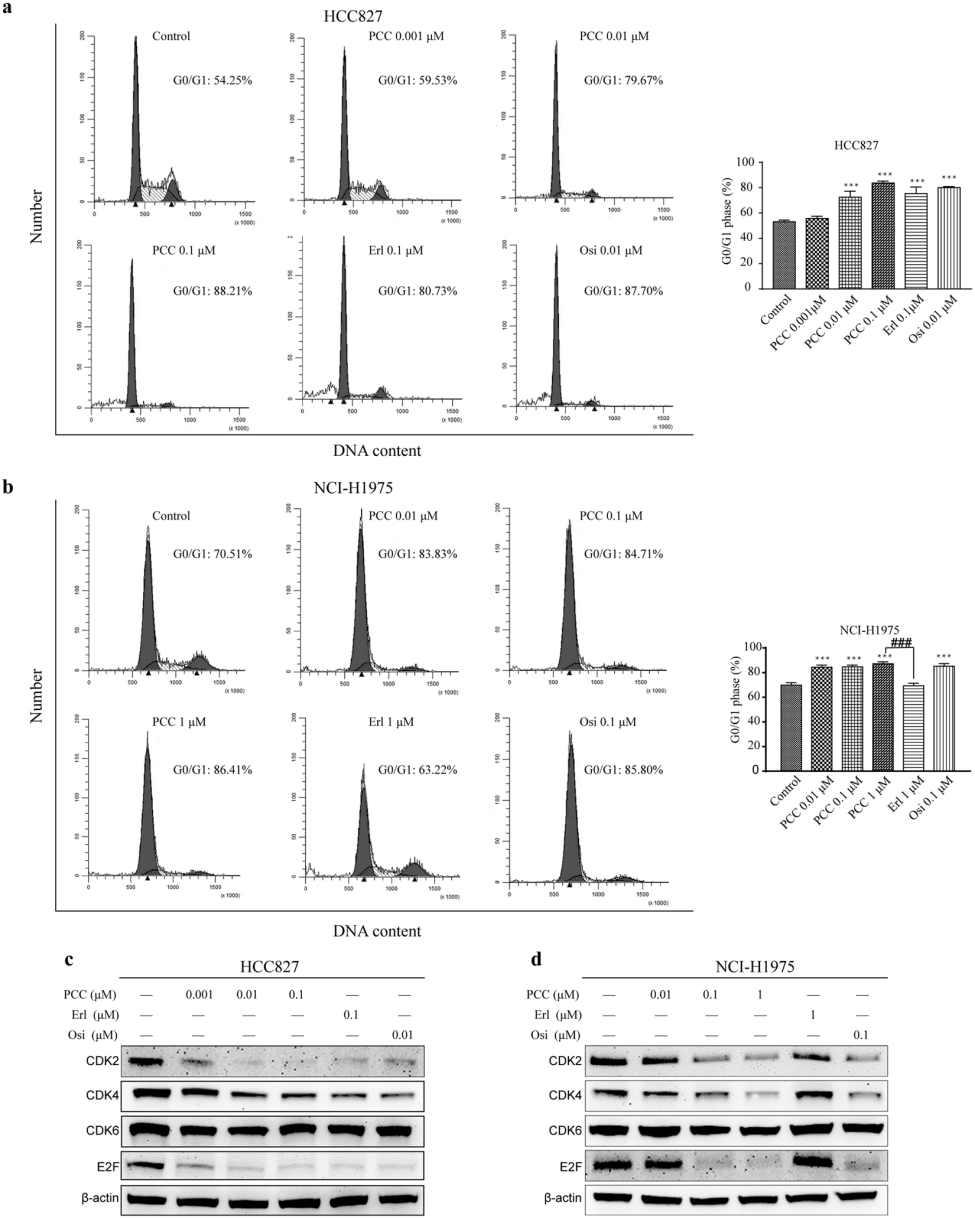
Effect of PCC0208027 on cell cycle progression. (**a**) HCC827 and (**b**) NCI-H1975 cells were fixed and stained with PI following treatment with PCC0208027, erlotinib, and osimertinib for 24 h. Cell cycle distribution was then detected using flow cytometry. PCC0208027 and osimertinib induced G0/G1 arrest in both HCC827 and NCI-H1975 cells, but erlotinib did not in NCI-H1975 cells. Columns, mean; bars, s.d. (n = 3). ^***^P < 0.001, compared to control group. ^###^*P* < 0.001, compared to erlotinib group. (**c**,**d**) HCC827 and NCI-H1975 cells were treated with various concentration of PCC0208027, erlotinib, and osimertinib for 24 h. The proteins associated with cell cycle were checked by western blot assay. PCC0208027 treatment significantly decreased the expression of CDK2, CDK4 and the nuclear transcription factor E2F. The full-length blots are presented in the Supplementary Materials. The gels were operated under the same experimental conditions in HCC827 and NCI-H1975 cells, respectively.

In order to evaluate the effect of PCC0208027 on cell cycle arrest in EGFR-driven NSCLC cells, we carried out flow cytometry analysis to quantitate changes in DNA content after 24 hours of treatment with different drugs. The results (Fig. 2(a,b)) showed that PCC0208027 significantly increased the number of cells in the G0/G1 phase in the treated group and showed significant dose-dependence in HCC827 and NCI-H1975 cells, while erlotinib only affected HCC827 cells. The differences between PCC0208027 and osimertinib were not statistically significant when the same doses were used in HCC827 and NCI-H1975 cells.

### PCC0208027 decreased the expression of key proteins associated with cell cycle

CDK2 and CDK4 are catalytic subunits of cyclin-dependent protein kinase (CDK) complexes. Their activities are controlled by the G1-S phase and are of vital importance in the G1-S transition. In order to further study the molecular mechanisms of G0/G1 arrest induced by PCC0208027 in HCC827 and NCI-H1975 cells, we employed western blotting to analyze changes in the expression level of key molecules that are associated with G0/G1 phase regulation in the cell cycle. As shown in Fig. 2(c,d), the expression of CDK2 and CDK4 in cells after 24 hours of PCC0208027 treatment was significantly inhibited and downstream E2F expression was drastically reduced.

### PCC0208027 inhibited phosphorylation of EGFR and downregulated downstream signals

**Figure 3:**
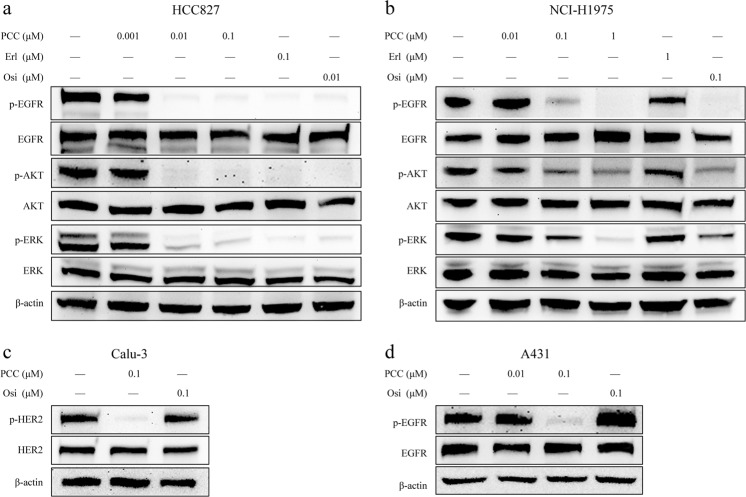
PCC0208027 inhibited phosphorylation of EGFR and depressed downstream signaling. Cells were treated with different concentrations of PCC0208027, erlotinib, or osimertinib for 24 h. Cells were lysed and proteins were analyzed by Western Blotting with the indicated antibodies. (**a**) Phosphorylation of EGFR and its downstream AKT and ERK key molecules were inhibited by PCC0208027, erlotinib, and osimertinib in HCC827 cells. (**b**) However, in NCI-H1975 cells, EGFR signaling was inhibited only by treatment with PCC0208027 and osimertinib. (**c**) Phosphorylation level of HER2 was inhibited obviously by PCC0208027 in Calu-3 cells. The full-length blots images are shown in the Supplementary Materials. The gels were operated under the same experimental conditions in each cell line. (**d**) In A431 cells with EGFR WT, PCC0208027 also showed potential inhibitory activity against phosphorylation of EGFR.

The EGFR Del E746-A750 HCC827 cells and the EGFR L858R/T790M double mutation NCI-H1975 cells are cell lines with EGFR drug-sensitive and -resistant mutations, respectively. In this study, we used the 2 aforementioned cell lines to examine the effect of PCC0208027 on phosphorylation of EGFR family members and downstream signaling in order to study the molecular mechanisms of PCC0208027 activity. The results (Fig. 3(a,b)) showed that PCC0208027 can inhibit EGFR in HCC827 and NCI-H1975 cells, as well as the phosphorylation levels of AKT, ERK, and other downstream signaling molecules. Additionally, erlotinib only affected HCC827 cells. Furthermore, PCC0208027 can inhibit dramatically phosphorylation levels of HER2 in Clue-3 cells and EGFR in A431 cells, as shown in Fig. 3(c,d).

### PCC0208027 showed effective anti-tumor activity in NSCLC xenograft models

**Figure 4:**
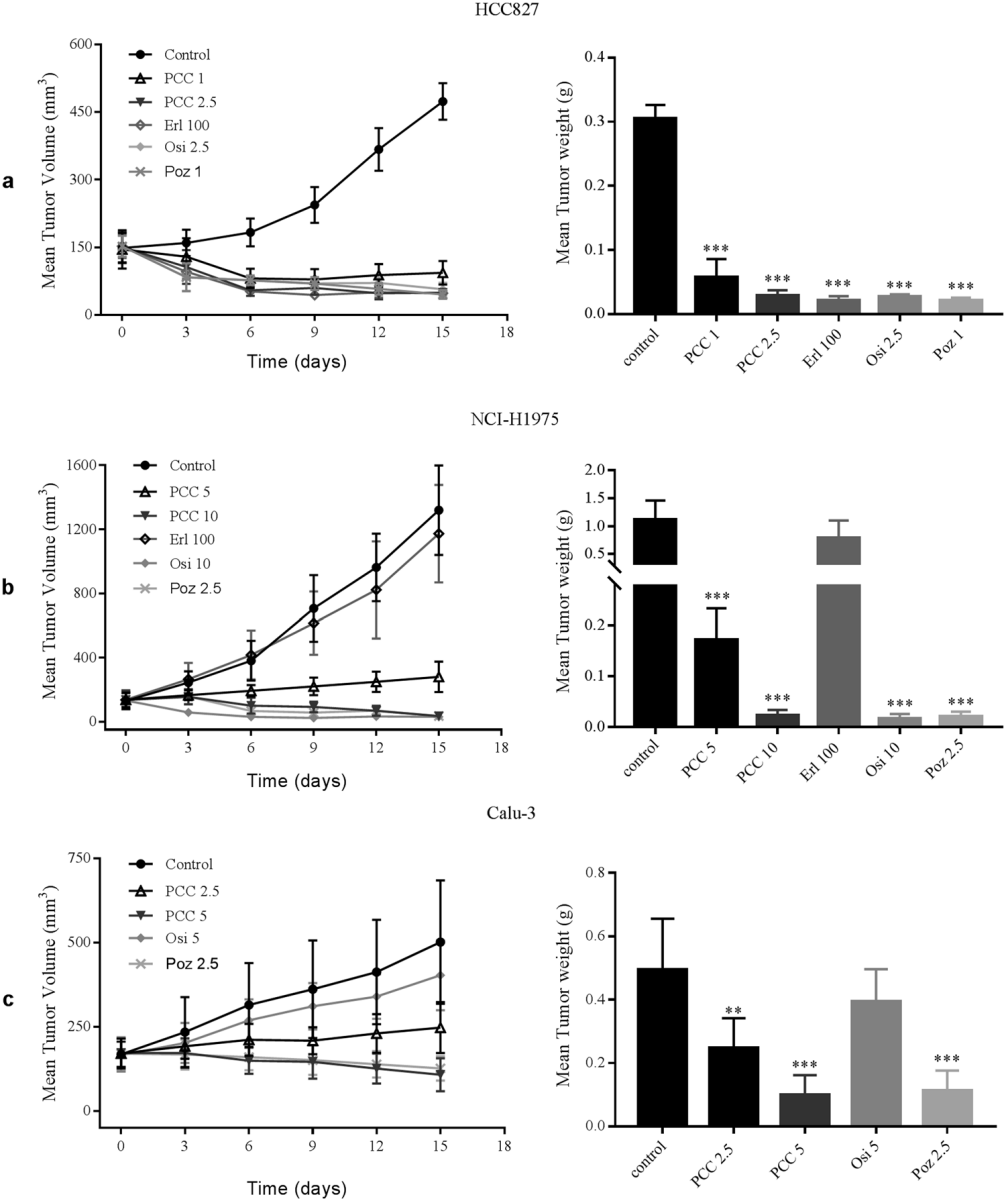
PCC0208027 produced robust anti-tumor effects in NSCLC tumor xenograft models. PCC0208027 inhibited tumor growth of HCC827, NCI-H1975, and Calu-3 xenograft models. HCC827, NCI-H1975, and Calu-3 cells (4 × 10^6^ cells/mouse) were implanted into the right flank of each animal. PCC0208027 was administered orally once daily for 15 days at different dose levels. (**a**) Tumor volume (left) and tumor weight (right) were satisfactorily inhibited in HCC827 tumor xenograft model after treatment with PCC0208027. One mouse died on day 14 in erlotinib group. (**b**) Tumor volume (left) and tumor weight (right) were effectively inhibited in NCI-H1975 tumor xenograft model after treatment with PCC0208027 while there was no effect and one mouse died on day 14 in the erlotinib group. (**c**) Tumor volume (left) and tumor weight (right) exhibited dose-dependent decreases after treatment with PCC0208027 in Calu-3 tumor xenograft model. Columns, mean; bars, s.d. (n = 3). ^*^P < 0.05, ^***^P < 0.001, compared to the control group.

In order to further examine the *in vivo* anti-tumor activity of PCC0208027, we used HCC827, NCI-H1975, and Calu-3 xenograft models to examine the effect of PCC0208027 on tumor growth and compared these effects with that of erlotinib, osimertinib and poziotinib. According to the results shown in Table 3 and Fig. 4, PCC0208027 exhibited persistent tumor suppressive activity at doses of 1–2.5 mg/kg/day on HCC827 xenografts. The maximum inhibition rate (IR) of PCC0208027 was 90.53% at 2.5 mg/kg/day, which was comparable to 100 mg/kg/day of erlotinib, 2.5 mg/kg/day of osimertinib and 1 mg/kg/day of poziotinib. In NCI-H1975 xenograft models, PCC0208027 exhibited excellent anti-tumor activity at doses of 5–10 mg/kg/day and showed similar inhibitory activity as osimertinib at identical doses. However, erlotinib did not inhibit tumor growth in NCI-H1975 xenograft models (*P* = 0.0899, *vs*. control group). In Calu-3 xenograft models, PCC0208027 significantly inhibited tumor growth at a dose of 5 mg/kg/day (*P* < 0.0001, *vs*. control group) while osimertinib did not show any significant inhibitory activity (*P* = 0. 0.5823, *vs*. control group).

**Table 3:** The anti-tumor activity of PCC0208027 in NSCLC xenograft models.

	Group	Dosage (mg/kg/day)	No. of animals (Begin/End)	Body weight (g)		IR (%)
				Begin	End	
HCC827	Con	—	6/6	17.48 ± 0.67	19.95 ± 0.88	
	PCC	1	6/6	17.67 ± 0.60	18.22 ± 0.91	81.24^***^
	PCC	2.5	6/6	17.63 ± 0.68	17.92 ± 0.77	90.53^***^
	Erl	100	6/5	17.65 ± 0.95	13.82 ± 0.75	93.20^***^
	Osi	2.5	6/6	17.57 ± 1.03	19.13 ± 0.78	91.19^***^
	Poz	1	6/6	17.55 ± 0.96	17.21 ± 0.90	93.25^***^
NCI-H1975	Con	—	6/6	16.50 ± 0.94	18.35 ± 0.90	
	PCC	5	6/6	16.68 ± 0.57	15.70 ± 0.58	84.61^***^
	PCC	10	6/6	16.40 ± 0.58	15.07 ± 0.65	97.98^***^
	Erl	100	6/5	16.63 ± 1.02	14.54 ± 1.10	29.78
	Osi	10	6/6	16.55 ± 0.82	16.23 ± 1.31	98.60^***^
	Poz	2.5	6/6	16.44 ± 0.69	14.62 ± 0.67	98.18^***^
Calu-3	Con	—	5/5	17.18 ± 0.97	18.48 ± 0.83	
	PCC	2.5	5/5	17.30 ± 0.37	16.94 ± 0.44	50.02^**^
	PCC	5	5/5	17.62 ± 0.77	16.56 ± 0.59	80.07^***^
	Osi	5	5/5	17.42 ± 0.82	18.24 ± 0.78	20.21
	Poz	2.5	5/5	17.72 ± 0.73	16.36 ± 0.92	77.16^***^

^**^P < 0.01, ^***^P < 0.001, compared to the control group.

According to the results shown in Table 3, in the HCC827 xenograft models, the body weight of animals in the erlotinib group decreased by more than 20% but the body weight of animals in the PCC0208027 and osimertinib groups showed varying degrees of increase. In the NCI-H1975 xenograft models, all drug treatment groups showed varying degrees of weight loss, with the exception of the control group. However, the body weight of animals in the PCC0208027 group decreased by less than 10%, indicating that the mice were able to tolerate doses of 1–10 mg/kg/day of PCC0208027 for 15 days. In addition, it is worth noting that 1 mouse died in the erlotinib groups in the HCC827 and H1975 xenograft models. In summary, PCC0208027 showed good inhibitory activities, regardless of the NSCLC tumor model, and exhibited a safe and tolerable drug profile in treated animals.

**Figure 5:**
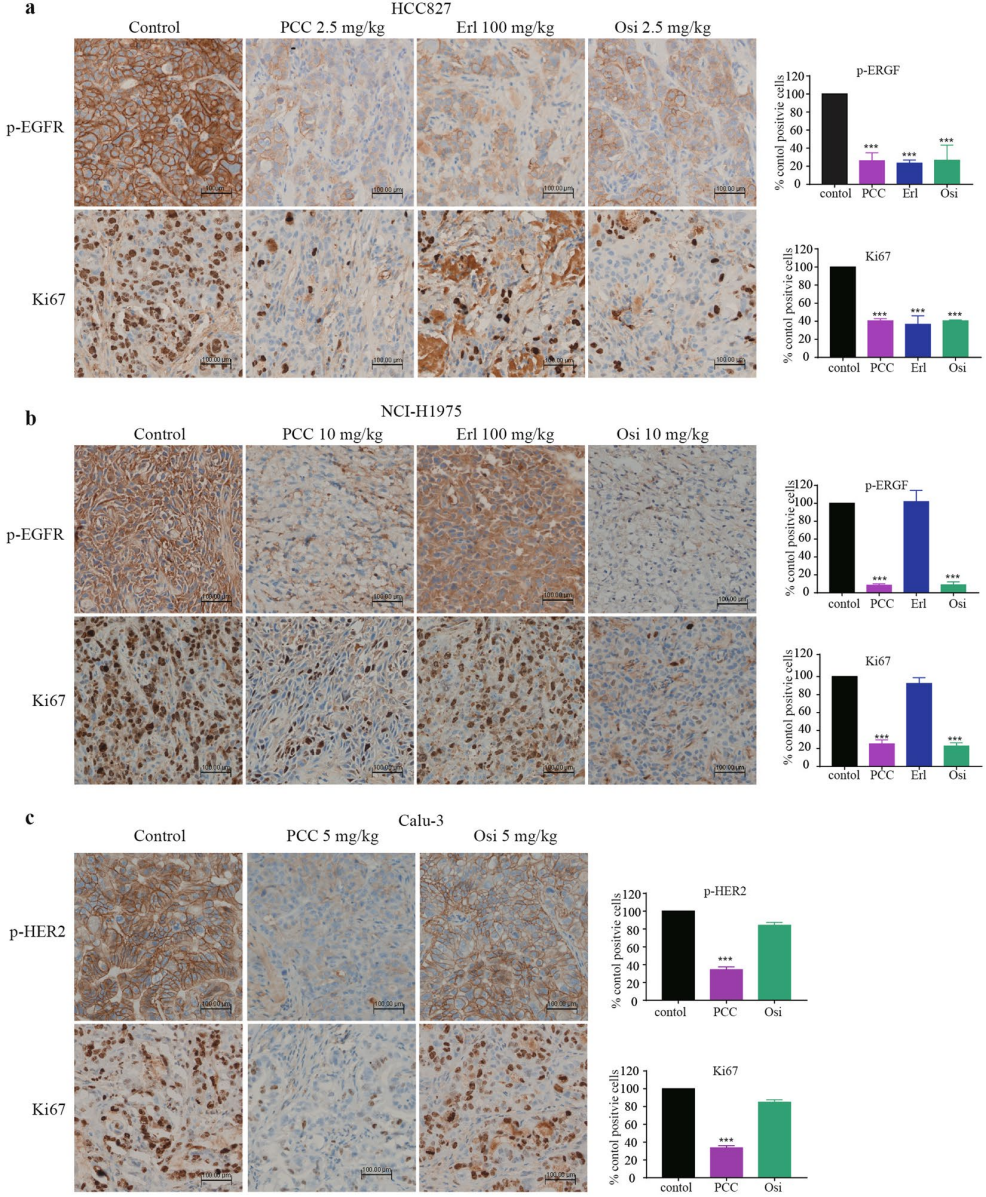
Mechanism of PCC0208027 anti-tumor activity in NSCLC tumor xenograft models. Ki67 and p-EGFR (or p-HER2) positive cells was observed in the tumor tissue (brown-colored objects) (200×). (**a**) PCC0208027, erlotinib, and osimertinib strongly decreased the number of p-EGFR and Ki67 positive cells in tumor tissues in comparison to the control group in HCC827 xenograft model. (**b**) PCC0208027 and osimertinib strongly decreased the number of p-EGFR and Ki67 positive cells in tumor tissues in NCI-H1975 xenograft model. (**c**) PCC0208027 significantly reduce the number of p-HER2 and Ki67 positive cells in tumor tissues in comparison to the control group in Calu-3 xenograft model. Columns, mean; bars, ^***^P < 0.001, compared to the control group.

Immunohistochemistry (IHC) analysis in animal tissues showed that the number of Ki67-positive cells in tumors from the PCC0208027 group was significantly reduced in comparison to the control group in HCC827 (Fig. 5(a)), NCI-H1975 (Fig. 5(b)) and Calu-3 (Fig. 5(c)) xenograft models. Additionally, PCC0208027 acted as an EGFR-TKI by significantly inhibiting EGFR and HER2 phosphorylation levels in tumor tissues. The results suggest that PCC0208027 can inhibit EGFR and HER2 phosphorylation and cell proliferation to significantly inhibit the growth of NSCLC tumors.

## Discussion

NSCLC is still a destructive disease with poor prognosis. As the early clinical symptoms of NSCLC are insidious and not apparent, patients are usually diagnosed with advanced disease. The 5-year survival rate of advanced NSCLC is usually very low, with survival rates of 13% and 1% for stage IIIC and IV, respectively^22^. Systemic treatment is the mainstay treatment for advanced NSCLC. Platinum-based chemotherapy and targeted therapy are often used in patients with advanced stage NSCLC. With the rapid development of precision medicine, mutations and aberrant expression of oncogenes that play important roles in the occurrence and development of NSCLC have been discovered. Therefore, treatment targeting driver mutations in oncogenes can inhibit tumor growth and good efficacy can be obtained in clinical practice. On this basis, selection of suitable targeted agents based on the molecular subtypes of NSCLC has become increasingly important. *EGFR* is the most common driver gene in the occurrence and development of NSCLC. EGFR-targeting TKIs have significantly changed the NSCLC treatment landscape. However, the benefits obtained are limited and drug resistance inevitably occurs. Therefore, NSCLC treatment is still a significant clinical challenge, and more work is required to develop more effective EGFR-targeting therapies.

Erlotinib, gefitinib, and afatinib are first-line therapies that mainly act on EGFR exon 19 deletion or exon 21 L858R mutations in NSCLC patients. However, diarrhea, rashes, and other adverse reactions are common^11^. In particular, patients who initially respond to treatment develop acquired drug resistance within 1–2 years of treatment, and EGFR T790M mutation is the most common mechanism for drug resistance^12–14^. Although osimertinib can overcome drug resistance due to the EGFR T790M mutation, osimertinib-treated patients also become drug resistant. The EGFR C797S mutation is the most common and most important mechanism for osimertinib resistance^15^. Although osimertinib was approved for NSCLC patients who are positive for drug-sensitive EGFR mutations due to its superior PFS, overall survival (OS) data in NSCLC patients is still immature^23^. In this study, the binding modes of PCC0208027 with EGFRC797S and HER2 exon 20 mutations could not be predicted, as there have been no available reports of co-crystal complexes. However, eye checking and critical residue modeling showed that PCC0208027 can bind to EGFR T790M and WT-HER2. Subsequently, we systematically evaluated the effect of PCC0208027 on different EGFR mutations using cell-free kinase assays, in addition to *in vitro* and *in vivo* studies. PCC0208027 exhibited significant inhibitory activity against drug-sensitive EGFR d746–750 and EGFR L858R mutations, and drug-resistant EGFR T790M and C797S mutations. The inhibitory effect of PCC0208027 on the drug-sensitive EGFR d746–750 and EGFR L858R mutations were comparable to that of erlotinib and osimertinib. The inhibitory effect of PCC0208027 on the EGFR T790M mutation was comparable to that of osimertinib. Kinase activity analysis demonstrated that PCC0208027 also had a good inhibitory effect on the osimertinib-resistant EGFR C797S mutation. However, this phenotype still requires further *in vitro* and *in vivo* validation.

Activation of alternative signaling pathways in receptor tyrosine kinase signaling is a common mechanism for resistance to targeted therapies. *HER2* amplification has been shown to be an alternative pathway activated to drive resistance to EGFR-TKIs^17^. Based on a published report, in NSCLC patients who are resistant to first-generation EGFR TKIs, *HER2* amplification can occur during acquisition of drug resistance in approximately 12% of patients, and is mutually exclusive with the EGFR T790M mutation^17^. Currently, there are no small molecule inhibitors approved that can target *HER2* amplification or mutations in NSCLC, and HER2-targeting treatment remains an urgent need for NSCLC patients with HER2 aberrations. HER2 is another member of the EGFR tyrosine kinase family, and has a tyrosine kinase domain although it does not have a specific ligand. However, it can easily form heterodimers with other EGFR family members to mediate downstream mitogenic signaling pathways^24^. As HER2 dissociates slowly from growth factors, its heterodimers have greater ligand affinity and specificity in comparison to other heterodimers. When compared to other family members, HER2 has stronger activity in activating downstream signaling pathways and stronger persistence. HER2 is a preferential binding partner for EGFR. Therefore, simultaneously targeting these two proteins or inhibiting heterodimer formation is expected to increase drug efficacy. A study showed that combination therapy with trastuzumab (anti-HER2 antibody) and cetuximab (anti-EGFR antibody) demonstrated good anti-tumor activity in gastric cancer PDX models^25^. Another study also showed that combining trastuzumab and cetuximab can inhibit EGFR/HER2 dimerization to combat trastuzumab-resistant gastric cancer^26^. These studies suggest that simultaneous targeting of EGFR/HER2 and their heterodimerization may be a novel treatment strategy. In this study, we carried out a systematic evaluation of the effect of PCC0208027 on HER2 overexpressing NSCLC. PCC0208027 demonstrated good HER2 inhibitory activities at the kinase, cellular, and animal levels.

EGFR and HER2 can form homodimers or heterodimers after activation to activate intracellular signaling pathways, such as RAS/RAF/MEK/ERK, PI3K/AKT/mTOR, Src kinase, and STAT transcription factors associated with tumorigenesis, cancer progression, and drug resistance^27,28^. In this study, we employed Western blotting to evaluate ERK and AKT phosphorylation levels after HCC827 and NCI-H1975 cells were treated with PCC0208027. Results showed that PCC0208027 acted as an EGFR inhibitor and significantly inhibited phosphorylation of ERK and AKT in downstream signaling pathways by inhibiting EGFR phosphorylation. IHC analysis of *ex vivo* tumor tissues also showed consistent results. Therefore, the anti-tumor activity of PCC0208027 was associated with the simultaneous inhibition of EGFR and HER2 activation and blockade of RAF/MEK/ERK and AKT/mTOR signaling pathways.

There are some limitations in this study. Firstly, kinase analysis showed that PCC0208027 had an inhibitory effect on the EGFR C797S mutation but this was not validated in cellular and *in vivo* animal models. Relevant studies are still ongoing. In addition, PCC0208027 showed significantly greater inhibitory activity than erlotinib and osimertinib on drug-sensitive EGFR mutations at the kinase level. However, this did not translate to significantly better results at the cellular and animal levels. This may be due to physical and chemical properties of PCC0208027 including membrane permeability, which requires further studies on structure-activity relationship, salt type, and production of this inhibitor.

In conclusion, *in vivo* and *in vitro* studies demonstrated that PCC0208027 had strong anti-tumor activity on NSCLC tumors with drug-sensitive EGFR mutations, drug-resistant EGFR mutations, and HER2 amplification. The primary mechanism of this anti-tumor activity is the simultaneous blockade of EGFR and HER2 activation, which downregulated downstream RAS/RAF/MEK/ERK and PI3K/AKT/mTOR pathways, resulting in G0/G1 arrest in cells. As a novel EGFR TKI that targets several EGFR mutations and HER2 amplification, it is worth conducting further research to understand PCC0208027 activity, in order to provide more feasible EGFR TKI options for NSCLC treatment.

## Methods

### Cell lines and culture

Human NSCLC cells NCI-H1975, HCC827, Calu-3 and A549were obtained from the Cell Culture Center of the Institute of Basic Medical Sciences, Chinese Academy of Medical Sciences. NCI-H1781 cell line was purchased from Cobioer Biosciences Co., Ltd. With the exception of Calu-3, all cell lines were cultured in RPMI-1640 medium (Gibco, USA) supplemented with 10% fetal bovine serum (FBS) (Gibco, USA). Calu-3 was maintained in Minimum Essential Medium (MEM) supplemented with 10% FBS (Gibco, USA), 5% 100 mM Sodium Pyruvate (Gibco, USA), and 5% Non-Essential Amino Acids Solution (NEAA; Gibco, USA). All cells were incubated at 37 °C with 5% CO2 in air.

### Chemicals and reagents

PCC0208027 and poziotinib were synthesized by Luye Pharma. Erlotinib and osimertinib were purchased from Selleckchem (Houston, USA).

Anti-EGFR (D38B1), anti-Phospho-EGFR (Tyr1068), anti-HER2/ErbB (D8F12), anti-Phospho-EGFR/ErbB (Tyr1221/1222) (6B12), anti-p44/42 MAPK (Erk1/2) (137F5), anti-Phospho-p44/42 MAPK (Erk1/2) (Thr202/Tyr204), anti-Akt (pan) C67E7, anti-Phospho-Akt (Ser473), CDK2 (78B2), CDK4 (DCS156), CDK6 (DCS83), and E2F-1 were obtained from Cell Signaling Technology (Beverly, MA). β-actin and the horseradish peroxidase (HRP)-conjugated secondary antibodies were purchased from Beyotime Biotechnology (Shanghai, China).

### *In vitro* kinase assay

We tested and analyzed the inhibitory effect of PCC0208027 against the 10 protein kinases, EGFR Wild Type, Her2 Wild Type, Her4 Wild Type, EGFR T790M, EGFR T790M/L858R, EGFR d746–750/T790M, EGFR C797S, EGFR L858R, EGFR d746–750 and EGFR C797S/T790M/L858R (obtained from Invitrogen (Shanghai) Co., Ltd.) and calculated its IC_50_. Various enzymes and biotin-labeled substrates were added to the test plates and incubated at room temperature for 15 minutes. Following that, ATP and the test compound was added, mixed, and incubated with the test plates. After completion of the reaction, the detection reagent is added to every well and incubated for 60 minutes before detection. Envision (PerkinElmer Inc.) was used to analyze the test plates and calculate the IC_50_ values. The IDBS XL fit module was used as the analysis module.

### Docking pipeline

Docking calculation was carried out utilizing CDOCKER program. The X-ray crystal of EGFR T790M was obtained from protein databank (PDB code: 4G5P) and used as the receptor. 4G5P is a crystal structure of epidermal growth factor receptor (T790M) complexed with afatinib^19^. PCC0208027 was sketched in ChemBioDraw 14.0 and converted into a 3D structure followed by local minimization using CHARMm force field. The resulting structure was used for the following docking simulations.

A 3D structure of WT-HER2-PCC0208027 was constructed by copying ligand coordinates to the substrate binding pocket of HER2 kinase. The X-ray structure of WT-HER2 homodimer in a complex with TAK-285 (PDB code: 3RCD) was obtained from Protein Data Bank and was reserved for docking-based studies^20^. First, protein geometry was optimized and checked by toolkit of DS3.0. PCC0208027 was converted into 3D structure followed by local minimization using CHARMm force field. Then, 10 replicas for each described compound were produced as a spherical scope with a diameter of 20 Å and centered on HER2 inhibitor adducts. Additionally, random conformation was limited to 10, simulated annealing methods were set to true, and other parameters were retained by default. Finally, ten best conformations were saved for further analysis and molecular docking.

### Cell growth inhibition assay

According to the previous report^29^, 5 × 10^3^ cells/100 μL were seeded in culture medium in every well in a 96-well plate for 24 hours. Then cells were exposed to different concentrations of PCC0208027, erlotinib, osimertinib, or poziotinib. After 72 h of treatment, MTT (Beyotime Biotechnology, China) was added at a final concentration of 5 mg/ml and incubated for 3 hours. Following that, DMSO was used to dissolve the formazan product and the Molecular Devices SpectraMax M5 microplate reader (Molecular Devices, USA) was used to measure absorbance at 570 nm and IC_50_ was calculated.

### Cell cycle analysis

As described previously^30^, 2 × 10^5^ cells were seeded in each well in 6-well plates and treated with different concentrations of PCC0208027, and erlotinib and osimertinib for 24 h. Following that, the cells were collected and rinsed once with PBS before overnight fixation with pre-cooled 70% ethanol at −20 °C. The fixed cells were washed twice with cold PBS and incubated in the dark at 37 °C with 10 μL RNAse A and 50 μg/mL PI staining solution for 30 minutes. The cell cycle was analyzed by flow cytometery and Modifit LT software (version 5.0, Verity Software House, USA).

### Western blot assay

After treatment with PCC0208027, erlotinib, or osimertinib for 24 h, cells were washed with PBS, and then lysed with RIPA buffer containing 1% PMSF on ice^31^. After centrifugation at 12000 g for 15 minutes, the supernatant was collected. The BCA reagent kit (Beyotime Biotechnology) was used to measure protein content. The cell extracts (50 μg protein) were loaded onto a 4–20% ExpressPlus™ PAGE pre-cast gel (GenScript, China) for electrophoresis before transferring to a PVDF membrane. The membranes were blocked with 5% skimmed milk (prepared using Tris-buffered saline containing 0.05% Tween-20) before incubation with primary antibodies at 4 °C overnight. The membranes were then incubated with HRP-conjugated secondary antibodies at room temperature. The Alphaview SA system and software (version 3.4.0, ProteinSimple, USA) was used for quantitative analysis of the optical density of the bands.

### Xenograft models

Xenograft models were established according to the method previously described^32^. Female BalB/c *nu/nu* nude mice (5–6-week-old, obtained from Vital River Laboratory Animal Technology Co., Ltd) were used for *in vivo* experiments in this study. HCC827, NCI-H1975, and Calu-3 cells were resuspended in serum-free culture medium and mixed in a 1:1 ratio with Matrigel (BD Biosciences, USA) before subcutaneous injection at the right scapula of each animal (4 × 10^6^ cells/mouse). The mice were randomized into groups when the average tumor volume reached 100–200 mm^3^. PCC0208027, erlotinib, osimertinib and poziotinib were dissolved in 10% Solutol^®^ HS 15 (sigma, USA). Different doses of PCC0208027, erlotinib, osimertinib, poziotinib or vehicle were given orally to mice every day. The inhibition rate (IR) of tumor growth was calculated using the following equation: IR (%) = (A − B)/A × 100, where A and B are the mean tumor weights of the vehicle control and treatment groups, respectively. All animal researches were performed in accordance with relevant guidelines and regulations approved by the the Ethics Committee of Shenyang Pharmaceutical University (No. 028 in 2017 for Animal Ethics Approval).

### Statistical analysis

Data are expressed as mean ± s.d. and analyzed using one-way ANOVA. Statistical analysis was performed using GraphPad Prism^®^ 7 (version 7.00, GraphPad Software, USA), and *P* < 0.05 was considered statistically significant.

## Supplementary information

PCC0208027, a novel tyrosine kinase inhibitor, inhibits tumor growth of NSCLC by targeting EGFR and HER2 aberrations.

## Data Availability

All the data about this present work can reasonably be requested from H. K. (email: liyuanboHK@163.com)
