# Supplementary material for: PCC0208027, a novel tyrosine kinase inhibitor, inhibits tumor growth of NSCLC by targeting EGFR and HER2 aberrations
Source: Sci Rep. 2019 Apr 5;9:5692. doi: 10.1038/s41598-019-42245-3 (PMC6451005; doi:10.1038/s41598-019-42245-3)

PCC0208027, a novel tyrosine kinase inhibitor, inhibits tumor growth of NSCLC by targeting EGFR and HER2 aberrations

Qiuju Dong[1], Pengfei Yu[3], Liang Ye[3], Jianzhao Zhang[2], Hongbo Wang[2], Fangxia Zou[2], Jingwei Tian[2, *] & Hiroshi Kurihara[1, *]

[1]School of Life Science and Biopharmaceutics, Shenyang Pharmaceutical University, Shenyang, P.R. China.

[2]School of Pharmacy, Key Laboratory of Molecular Pharmacology and Drug Evaluation (Yantai University), Ministry of Education, Collaborative Innovation Center of Advanced Drug Delivery System and Biotech Drugs in Universities of Shandong, Yantai University, Yantai, P.R. China.

[3]School of Pharmacy, Binzhou Medical University, Yantai, P.R. China.

Correspondence Authors: Hiroshi Kurihara (email: liyuanboHK@163.com) or Jingwei Tian (email: Tianjinwei@luye.com)

In some cases, 2 membranes were detected at the same time, and the irrelevant membrane in the pictures have been mosaicated in this file. The full-length blots used in the article have not been modified.

Figure 2(c): HCC827 CDK2
From left to right: Control, PCC 0.001, PCC 0.01, PCC 0.1, Erl 0.1, Osi 0.01 (μM)

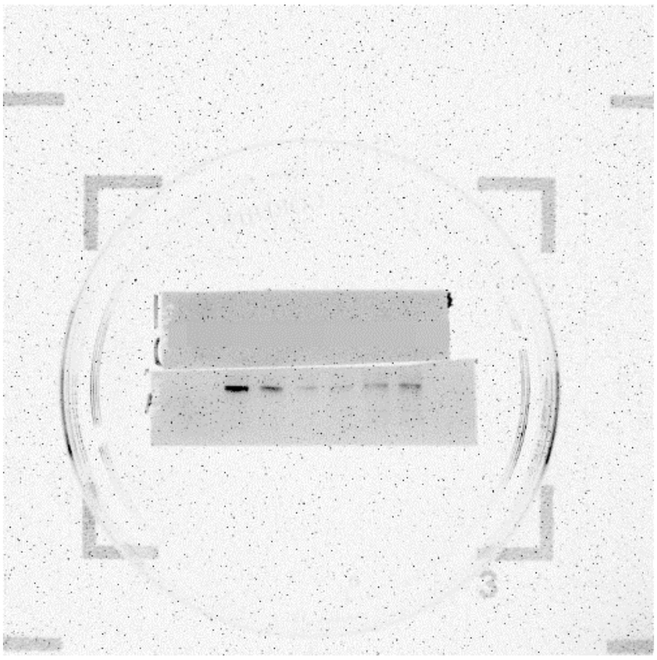

Figure 2(c): HCC827 CDK4
From left to right: Control, PCC 0.001, PCC 0.01, PCC 0.1, Erl 0.1, Osi 0.01 (μM)

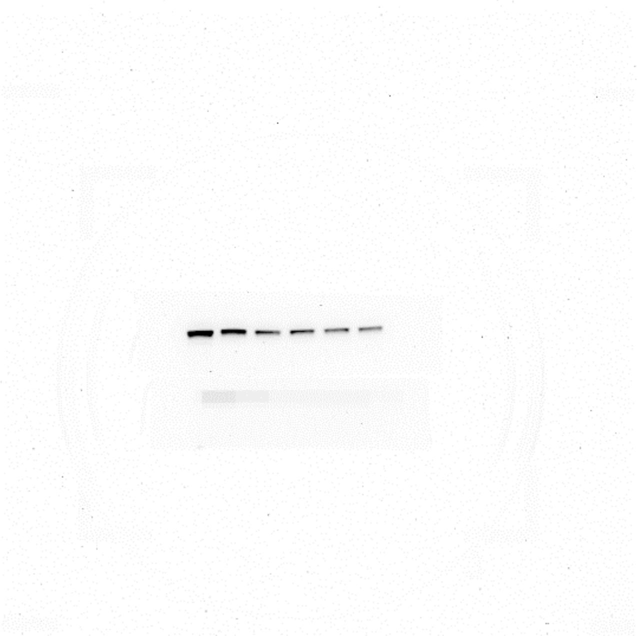

Figure 2(c): HCC827 CDK6

From left to right: Control, PCC 0.001, PCC 0.01, PCC 0.1, Erl 0.1, Osi 0.01 (μM)

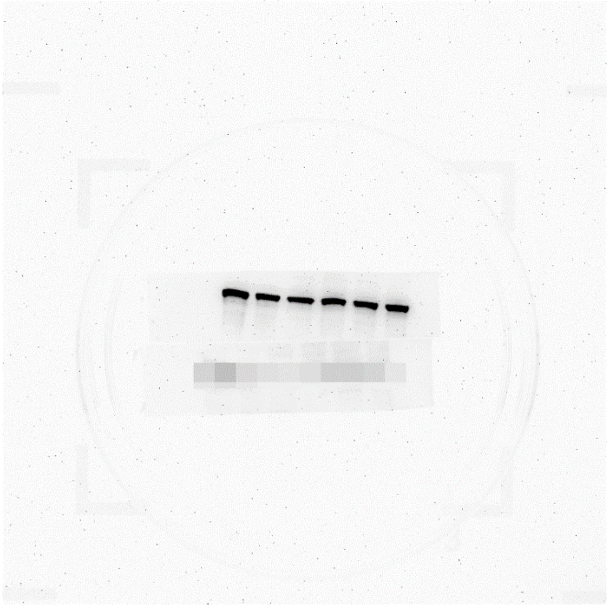

Figure 2(c): HCC827 E2F

From left to right: Control, PCC 0.001, PCC 0.01, PCC 0.1, Erl 0.1, Osi 0.01 (μM)

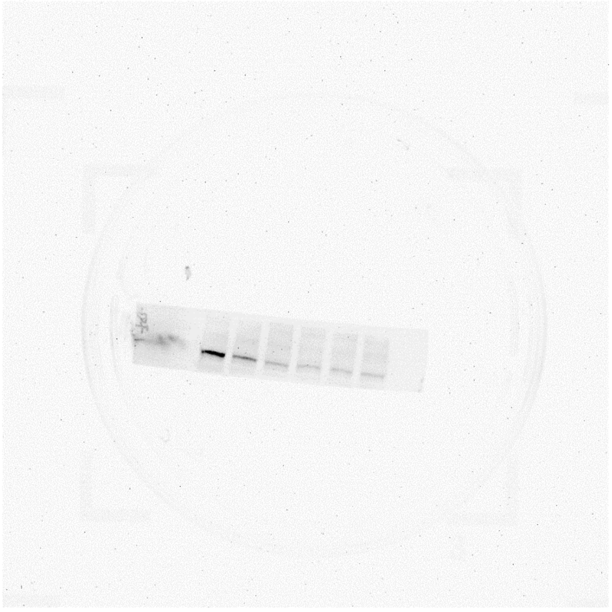

Figure 2(c): HCC827 β-actin

From left to right: Control, PCC 0.001, PCC 0.01, PCC 0.1, Erl 0.1, Osi 0.01 (μM)

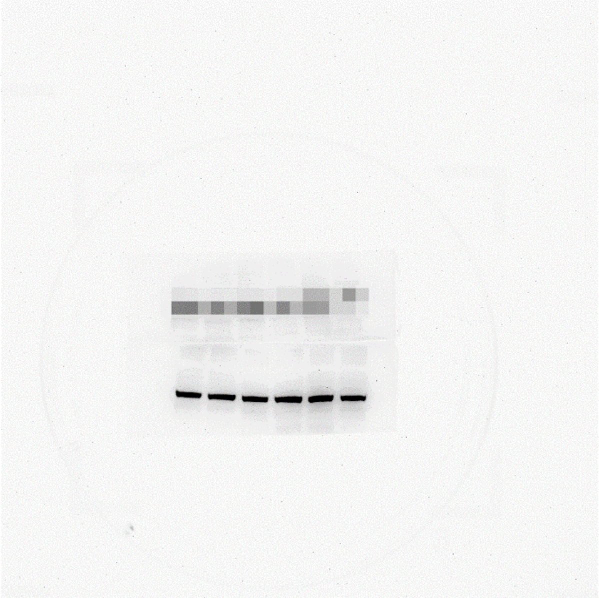

Figure 2(c): NCI-H1975 CDK2
From left to right: Control, PCC 0.01, PCC 0.1, PCC 1, Erl 1, Osi 0.1 (μM)

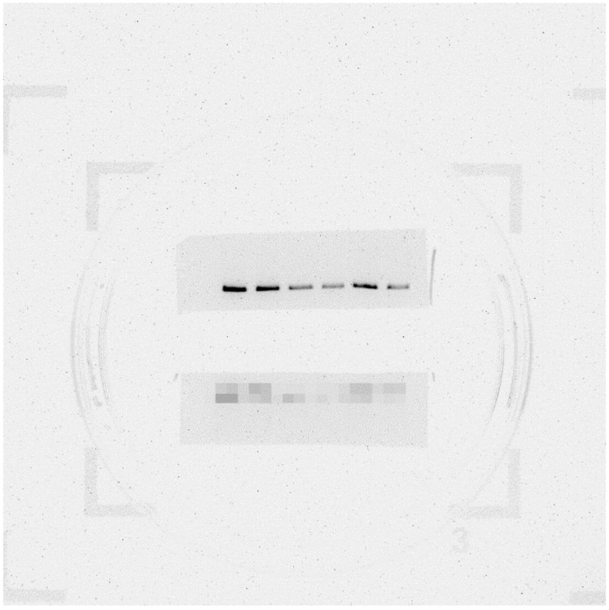

Figure 2(c): NCI-H1975 CDK4

From left to right: Control, PCC 0.01, PCC 0.1, PCC 1, Erl 1, Osi 0.1 (μM)

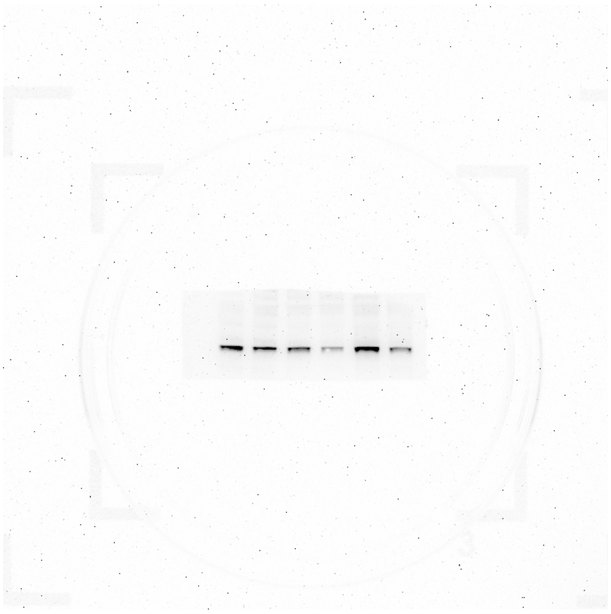

Figure 2(c): NCI-H1975 CDK6

From left to right: Control, PCC 0.01, PCC 0.1, PCC 1, Erl 1, Osi 0.1 (μM)

Figure 2(c): NCI-H1975 E2F

From left to right: Control, PCC 0.01, PCC 0.1, PCC 1, Erl 1, Osi 0.1 (μM)

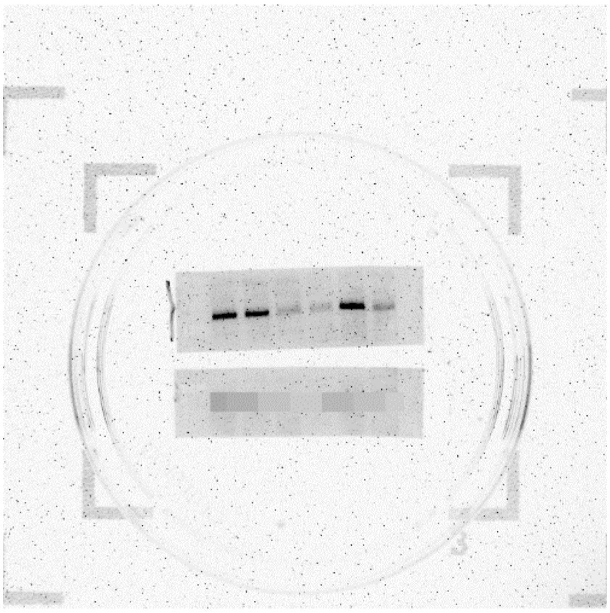

Figure 2(c): NCI-H1975 β-actin

From left to right: Control, PCC 0.01, PCC 0.1, PCC 1, Erl 1, Osi 0.1 (μM)

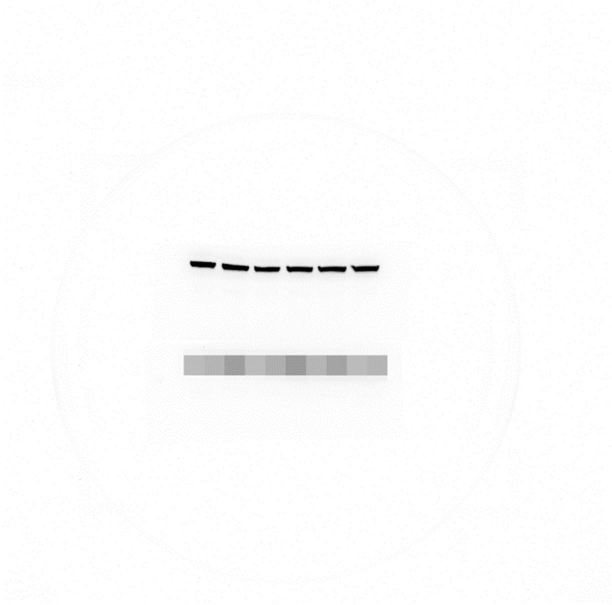

Figure 3(a): HCC827 pEGFR

From left to right: Control, PCC 0.001, PCC 0.01, PCC 0.1, Erl 0.1, Osi 0.01 (μM)

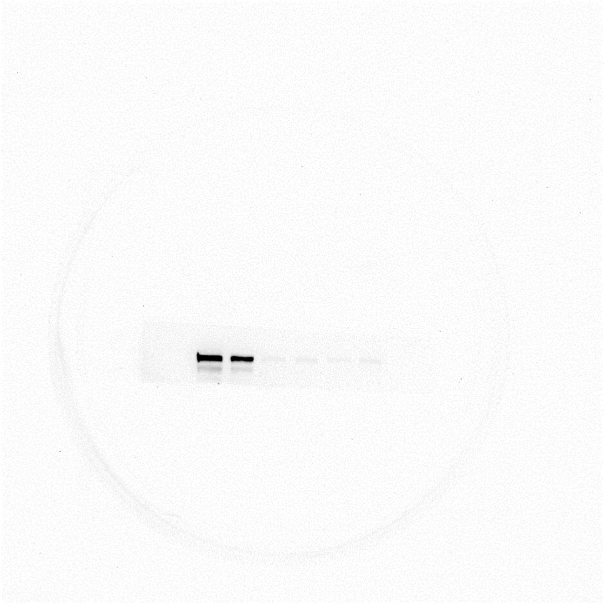

Figure 3(a): HCC827 EGFR

From left to right: Control, PCC 0.001, PCC 0.01, PCC 0.1, Erl 0.1, Osi 0.01 (μM)

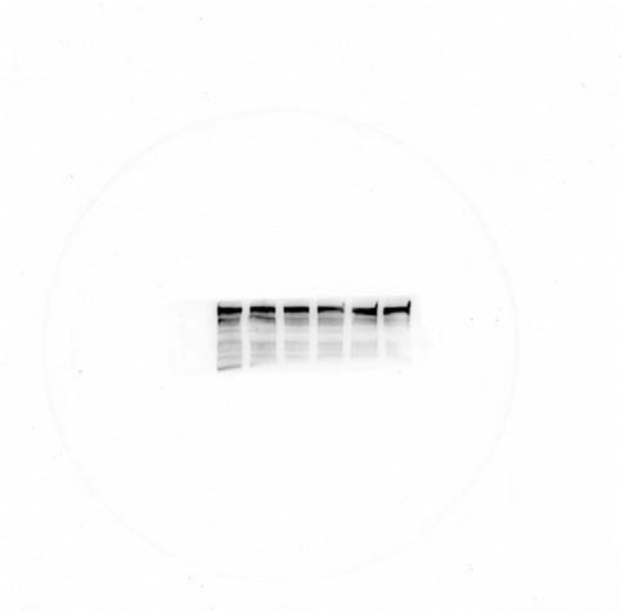

Figure 3(a): HCC827 pAKT
From left to right: Control, PCC 0.001, PCC 0.01, PCC 0.1, Erl 0.1, Osi 0.01 (μM)

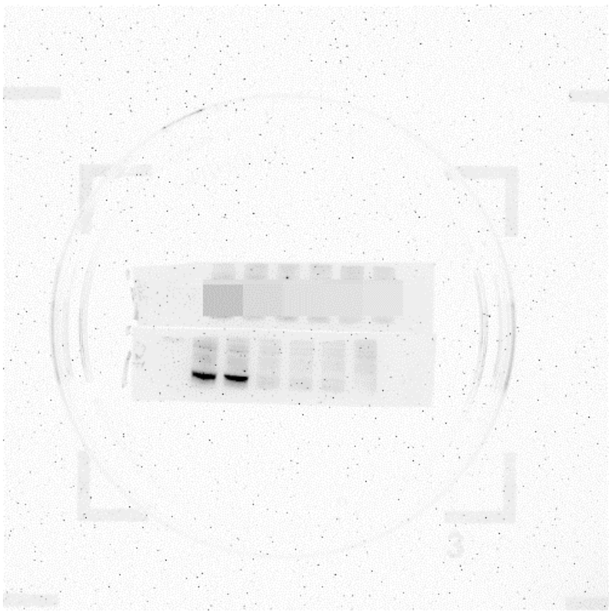

Figure 3(a): HCC827 AKT
From left to right: Control, PCC 0.001, PCC 0.01, PCC 0.1, Erl 0.1, Osi 0.01 (μM)

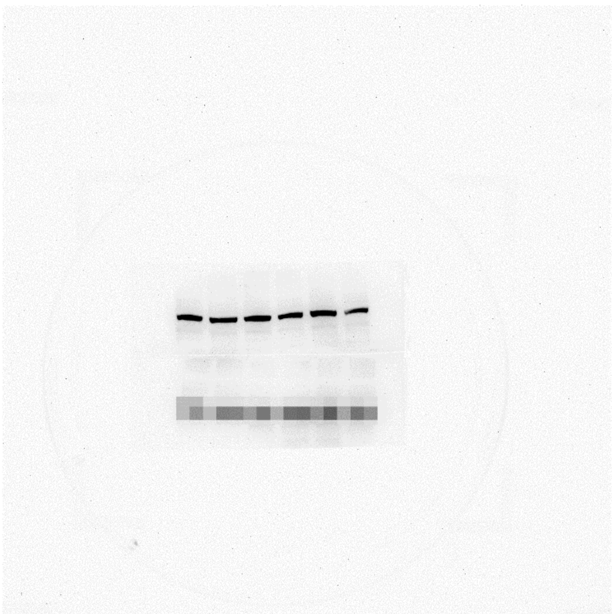

Figure 3(a): HCC827 pERK
From left to right: Control, PCC 0.001, PCC 0.01, PCC 0.1, Erl 0.1, Osi 0.01 (μM)

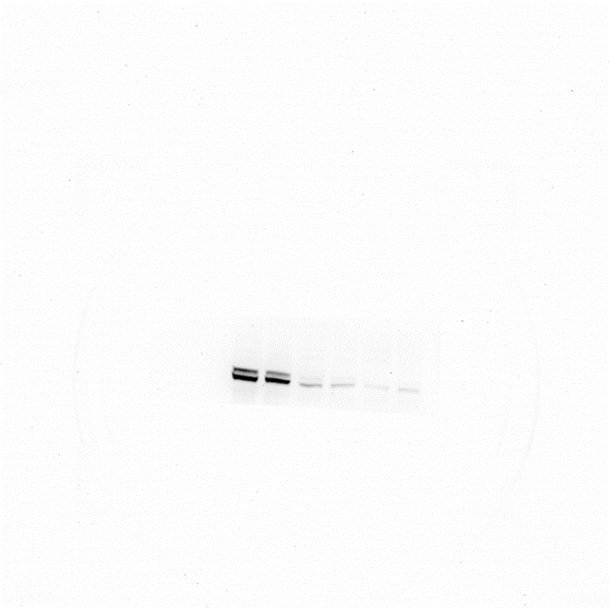

Figure 3(a): HCC827 ERK
From left to right: Control, PCC 0.001, PCC 0.01, PCC 0.1, Erl 0.1, Osi 0.01 (μM)

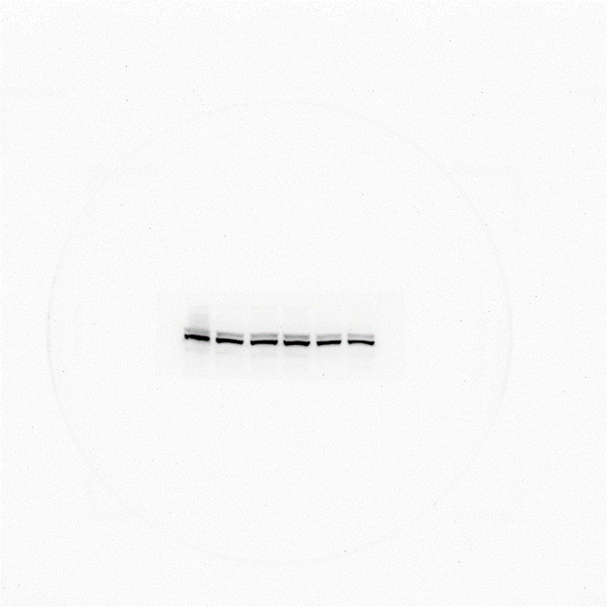

Figure 3(a): HCC827 β-actin

From left to right: Control, PCC 0.001, PCC 0.01, PCC 0.1, Erl 0.1, Osi 0.01 (μM)

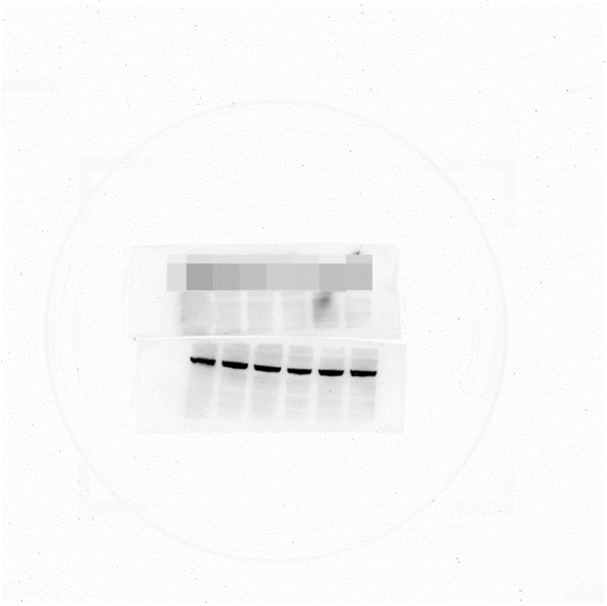

Figure 3(b): NCI-H1975 pEGFR

From left to right: Control, PCC 0.01, PCC 0.1, PCC 1, Erl 1, Osi 0.1 (μM)

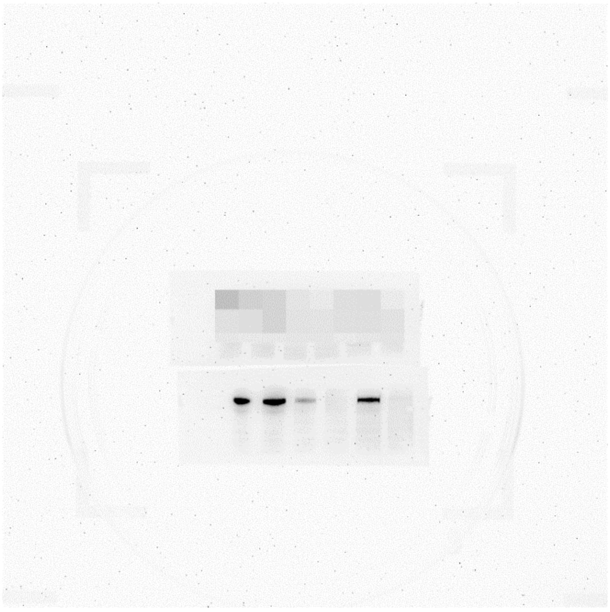

Figure 3(b): NCI-H1975 EGFR

From left to right: Control, PCC 0.01, PCC 0.1, PCC 1, Erl 1, Osi 0.1 (μM)

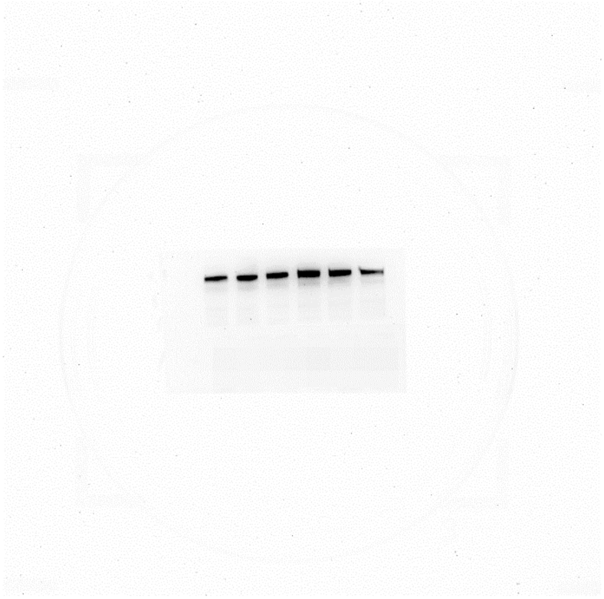

Figure 3(b): NCI-H1975 p-AKT

From left to right: Control, PCC 0.01, PCC 0.1, PCC 1, Erl 1, Osi 0.1 (μM)

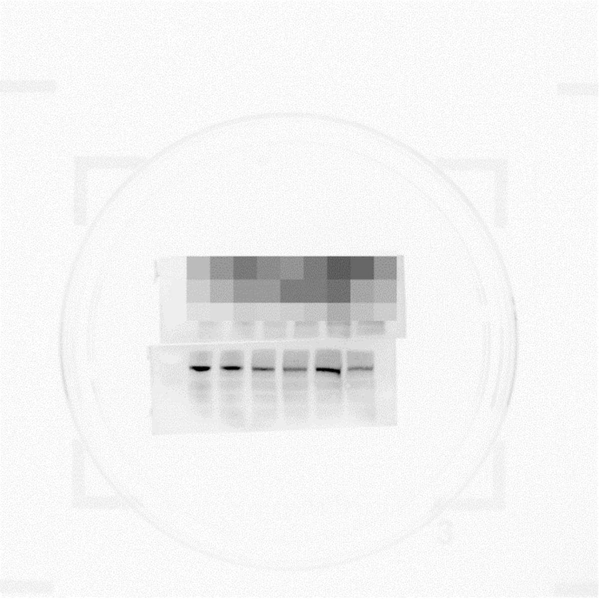

Figure 3(b): NCI-H1975 AKT

From left to right: Control, PCC 0.01, PCC 0.1, PCC 1, Erl 1, Osi 0.1 (μM)

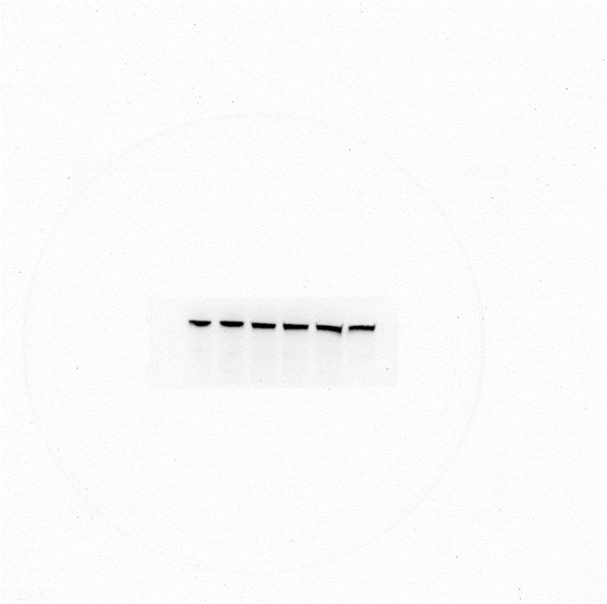

Figure 3(b): NCI-H1975 pERK

From left to right: Control, PCC 0.01, PCC 0.1, PCC 1, Erl 1, Osi 0.1 (μM)

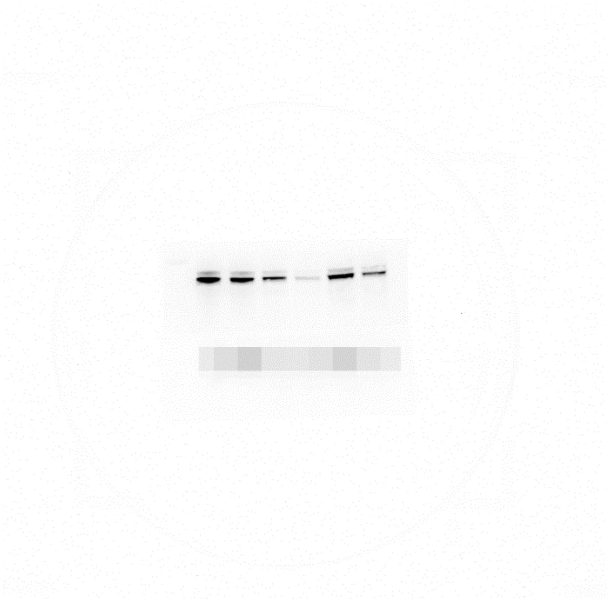

Figure 3(b): NCI-H1975 ERK

From left to right: Control, PCC 0.01, PCC 0.1, PCC 1, Erl 1, Osi 0.1 (μM)

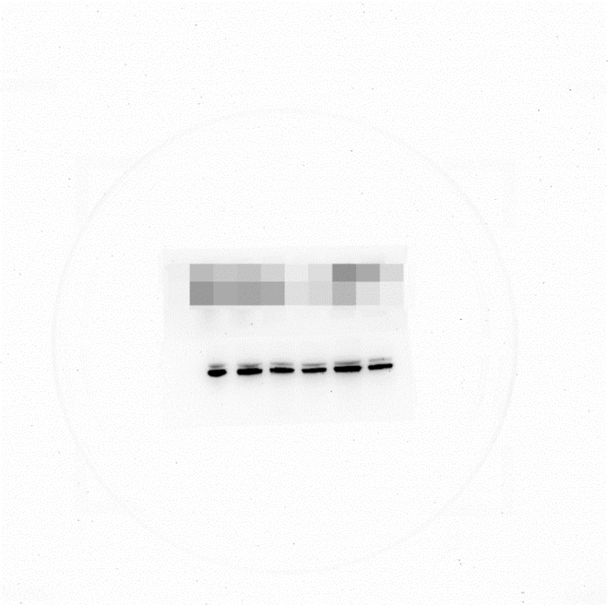

Figure 3(b): NCI-H1975 β-actin

From left to right: Control, PCC 0.01, PCC 0.1, PCC 1, Erl 1, Osi 0.1 (μM)

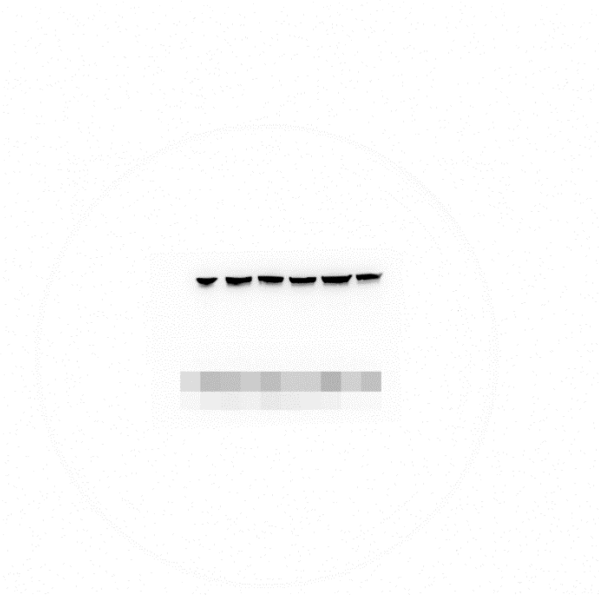

Figure 3(c): Calu3 p-Her2
From left to right: Control, PCC 0.1μM, Osi 0.1μM

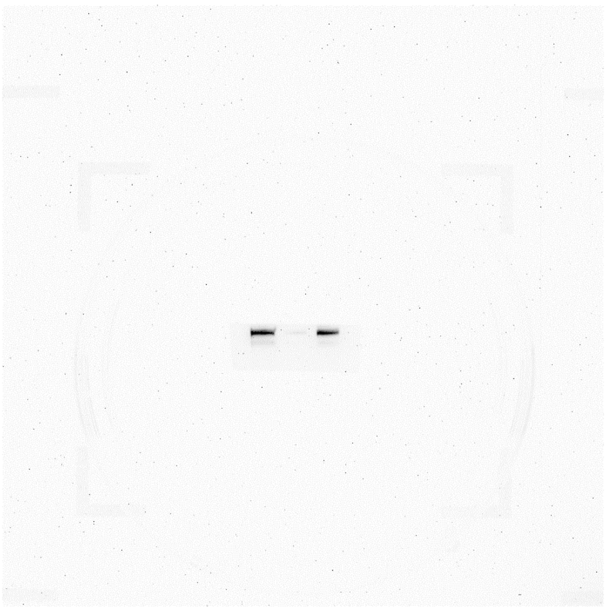

Figure 3(c): Calu3 Her2
From left to right: Control, PCC 0.1μM, Osi 0.1μM

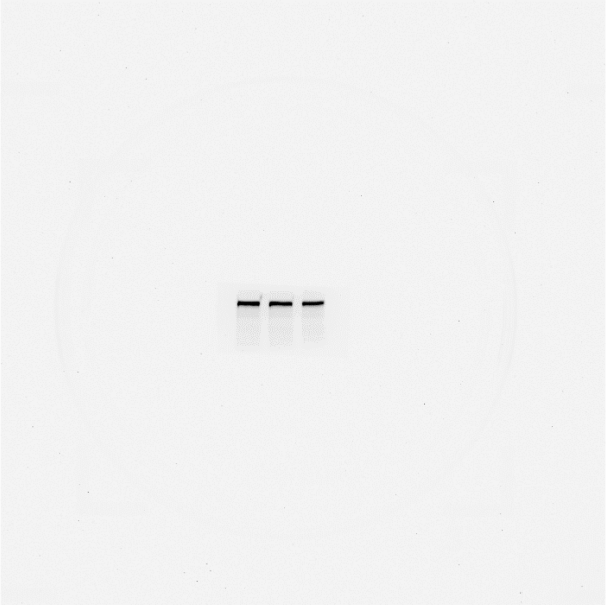

Figure 3(c): Calu3 β-actin

From left to right: Control, PCC 0.1μM, Osi 0.1μM

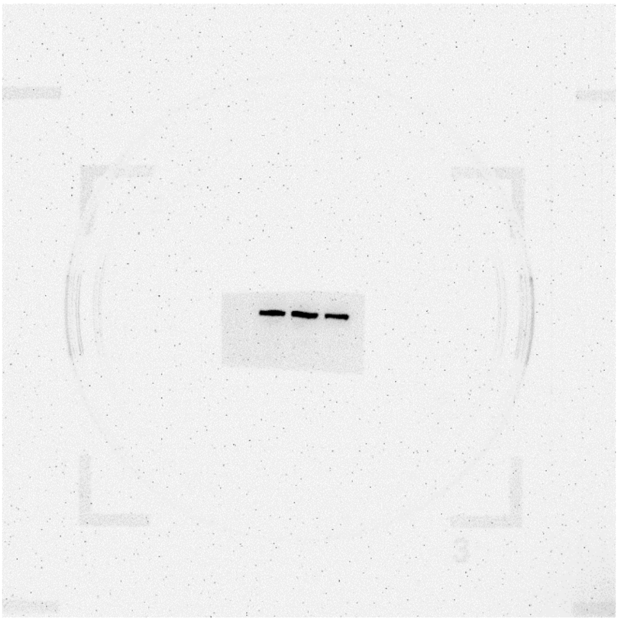

Figure 3(d): A431 p-EGFR

From left to right: Control, PCC 0.01μM, PCC 0.1μM, Osi 0.1μM, other sample

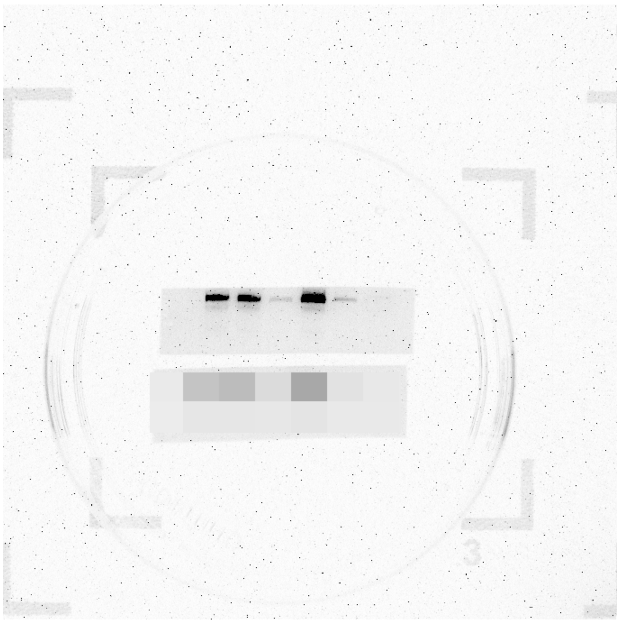

Figure 3(d): A431 EGFR

From left to right: Control, PCC 0.01μM, PCC 0.1μM, Osi 0.1μM, other sample

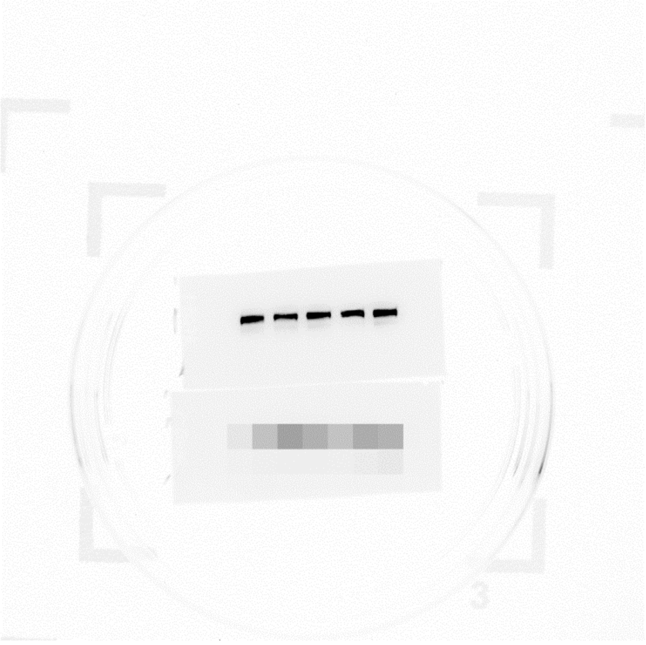

Figure 3(d): A431β-actin

From left to right: Control, PCC 0.01μM, PCC 0.1μM, Osi 0.1μM, other sample

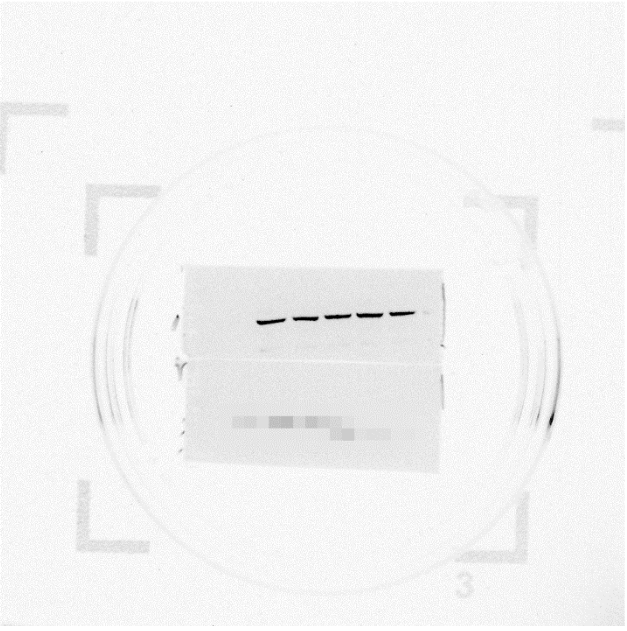

Supplement: Supplementary file 1 — PCC0208027, a novel tyrosine kinase inhibitor, inhibits tumor growth of NSCLC by targeting EGFR and HER2 aberrations. [file 41598_2019_42245_MOESM1_ESM.pdf]
